# Supplementary material for: Neuromorphic intermediate representation: A unified instruction set for interoperable brain-inspired computing
Source: Nat Commun. 2024 Sep 16;15:8122. doi: 10.1038/s41467-024-52259-9 (PMC11405706; doi:10.1038/s41467-024-52259-9)
Supplement: Supplementary file 1 — Supplementary Information [file 41467_2024_52259_MOESM1_ESM.pdf]

Supplementary Information Neuromorphic Intermediate Representation: A Unified Instruction Set for Interoperable Brain-Inspired Computing

Jens E. Pedersen *†[1], Steven Abreu†[2,3], Matthias Jobst[4,5], Gregor Lenz[6], Vittorio Fra[7], Felix Christian Bauer[8], Dylan Richard Muir[8], Peng Zhou[9], Bernhard Vogginger[4], Kade Heckel[10], Gianvito Urgese[7], Sadasivan Shankar[11,12], Terrence C. Stewart[13], Sadique Sheik[8], and Jason K. Eshraghian[14]

[1]KTH Royal Institute of Technology, Sweden
[2]CogniGron Center, University of Groningen, Netherlands
[3]Bernoulli Institute, University of Groningen, Netherlands
[4]Technische Universität Dresden, Germany
[5]Centre for Tactile Internet with Human-in-the-Loop, Dresden, Germany
[6]Neurobus, Toulouse, France
[7]Politecnico di Torino, Italy
[8]SynSense, Zurich, Switzerland
[9]LuxiTech Co. Ltd., Shenzhen, China
[10]University of Cambridge, UK
[11]Stanford University, USA
[12]SLAC National Laboratory, Menlo Park, CA, USA
[13]National Research Council Canada
[14]University of California, Santa Cruz, USA

July 2024

A Neuron model parameterizations

We now turn to the implementation details of NIR equations across the different platforms, with a particular focus on leaky integrate-and-fire dynamics from experiments 1 and 3 in the main paper. We note that the idealized reset can be discretized by either a hard reset to θ_{thr} or a "soft" subtractive reset that retains the residual from a possible overshoot:

$$\text{Hard reset} \qquad v_{t+1} = \begin{cases} v_t & v_t < \theta_{\text{thr}} \\ 0 & v_t \geqslant \theta_{\text{thr}} \end{cases} \tag{1}$$

$$\text{Subtractive reset} \qquad v_{t+1} = \begin{cases} v_t & v_t < \theta_{\text{thr}} \\ v_t - \theta_{\text{reset}} & v_t \geqslant \theta_{\text{thr}} \end{cases} \tag{2}$$

*Corresponding author: jeped@kth.se
†equal contribution

The differences in the discretization of spiking neuron dynamics are detailed below; equations for Spyx are not listed, as they follow the same implementation as snnTorch.

A.1 LIF

NIR The leaky integrate-and-fire model in NIR is given by:

$$\tau \dot{v} = v_{\text{leak}} - v + R\ i \tag{3}$$

where τ is the time constant, v_{leak} is the membrane leak, R is the resistance and $i(t)$ is the input current. Discretized using explicit Euler's method, this turns into:

$$v(t+1) = \left(1 - \frac{dt}{\tau}\right) v(t) + \frac{dt}{\tau} v_{\text{leak}} + \frac{dt}{\tau} R\ i(t) \tag{4}$$

where dt is the discretization timestep.

Exact solution for constant input current The course of the membrane potential v can be exactly calculated for a constant input current $i = i_0 = \text{const.}$ and initial condition $v(0) = v_0$ [1, eq. 1.7]:

$$v(t) = v_{\text{leak}} + Ri_0 \left(1 - \exp\left(-\frac{t}{\tau}\right)\right) + v_0 \exp\left(-\frac{t}{\tau}\right) \tag{5}$$

Lava-dl In lava-dl, we used the CuBa-LIF model as:

$$u(t) = (1 - \alpha_u) u(t-1) + i(t) \tag{6}$$

$$v(t) = (1 - \alpha_v) v(t-1) + u(t) \tag{7}$$

with $\alpha_u = 1$ to effectively turn it into a LIF model:

$$v(t) = (1 - \alpha_v) v(t-1) + i(t) \tag{8}$$

with

$$\alpha_v = \frac{dt}{\tau_{\text{mem}}} \tag{9}$$

With the constraints

$$R \frac{dt}{\tau_{\text{mem}}} = 1 \tag{10}$$

$$v_{\text{leak}} \frac{dt}{\tau_{\text{mem}}} = 0 \tag{11}$$

One may implement the first constraint (R) by hard-coding it into a linear layer, or into the voltage threshold. One may implement v_{leak} through a bias term, but this was not done in our experiments.

Norse The LIF model in Norse lacks the resistivity term, which we compensate for by scaling the input

$$v(t+1) = v(t) + \frac{dt}{\tau} \left(v_{leak} - v(t) + i\right) \tag{12}$$

snnTorch The LIF model in snnTorch is given by:

$$v(t+1) = \beta v(t) + i(t+1) \tag{13}$$

where β is the membrane potential decay rate. We have:

$$\beta = 1 - \frac{dt}{\tau} \tag{14}$$

And the constraints are:

$$R\frac{dt}{\tau} = 1 \tag{15}$$

$$v_{\text{leak}}\frac{dt}{\tau} = 0 \tag{16}$$

However, we can use a linear layer before the LIF in simulation to effectively implement the resistance term. The weight matrix of this linear layer is given by:

$$W_{ij} = \begin{cases} R\frac{dt}{\tau} & \text{if } i = j \\ 0 & \text{otherwise} \end{cases} \tag{17}$$

SpiNNaker2 In py-spinnaker2 [2] the LIF model is given by:

$$v(t) = \alpha_{\text{decay}} v(t-1) + i(t) + i_{\text{offset}} \tag{18}$$

Two modes for the translation between NIR and SpiNNaker2 are supported:

In Exponential-Euler mode, the decay factor α_{decay} is calculated such that in case without input ($i = 0$ and $i_{\text{offset}} = 0$) and with $v_{\text{leak}} = 0$ the voltage exactly matches the analytical expression for continuous-time dynamics:

$$v(t+dt) = \alpha_{\text{decay}} v(t) \stackrel{!}{=} v(t) \exp\left(-\frac{dt}{\tau}\right) \tag{19}$$

$$\Rightarrow \alpha_{\text{decay}} = \exp\left(-\frac{dt}{\tau}\right) \tag{20}$$

The SpiNNaker2 threshold Θ is scaled to cope for the non-exisiting resistance variable:

$$\Theta = \frac{\theta_{\text{thr}}}{\left(1 - \exp(-\frac{dt}{\tau})\right) R} \tag{21}$$

In Forward-Euler mode we match the discrete Euler update of the LIF model (Eq. 4) so that decay factor and threshold become:

$$\alpha_{\text{decay}} = 1 - \frac{dt}{\tau} \tag{22}$$

$$\Theta = \theta_{\text{thr}} \frac{\tau}{dtR} \tag{23}$$

In both modes, the offset current i_{offset} is used to consider the parameter v_{leak} and the bias i_{bias} from incoming `Affine` or `Conv2D` nodes:

$$i_{\text{offset}} = \frac{v_{\text{leak}}}{R} + i_{\text{bias}} \tag{24}$$

Nengo The default LIF model in Nengo's CPU implementation is identical to the Exponential-Euler model for SpiNNaker2 described above, except that it has a fixed threshold Θ. To compensate for this we adjust the gain on each neuron (a multiplicative factor applied to all inputs).

Rockpool The LIF model in Rockpool is a time-discretized neuron membrane, with difference or update equations

$$V_{mem}[n] = \alpha V_{mem}[n-1]$$
$$V_{mem}[n] = V_{mem}[n] + S_{in}[n] + b + \sigma\zeta[n]$$
$$V_{mem}[n] > \theta_{thr} \rightarrow S_{out}[n] = 1$$
$$V_{mem}[n] = V_{mem}[n] - \theta$$

where $\alpha = \exp(-dt/\tau_{mem})$. Here V_{mem} is the floating-point membrane potential state; S_{in} and S_{out} are the incident and output spike trains respectively; $\sigma\zeta[n]$ is a scaled white noise process with mean 0 and std. dev. σ; b is a bias current and θ is the firing threshold. We have neglected neuron indices in the above equations; all parameters are independently defined per neuron in Rockpool.

A.2 CuBa-LIF

NIR The current-based LIF model in NIR is given by:

$$\tau_{\text{syn}}\dot{u} = -u + w_{\text{in}}i \tag{25}$$
$$\tau_{\text{mem}}\dot{v} = v_{\text{leak}} - v + Ru \tag{26}$$

where $u(t)$ is the synaptic current, τ_{mem} the membrane time constant, τ_{syn} the synaptic time constant, and w_{in} the scaling factor of the current input $i(t)$. Discretized:

$$u(t+1) = \left(1 - \frac{dt}{\tau_{\text{syn}}}\right)u(t) + w_{\text{in}}\frac{dt}{\tau_{\text{syn}}}i(t+1) \tag{27}$$
$$v(t+1) = \left(1 - \frac{dt}{\tau_{\text{mem}}}\right)v(t) + \frac{dt}{\tau_{\text{mem}}}v_{\text{leak}} + R\frac{dt}{\tau_{\text{mem}}}u(t) \tag{28}$$

Lava-dl In lava-dl, the model is:

$$u(t) = (1-\alpha_u)u(t-1) + i(t) \tag{29}$$
$$v(t) = (1-\alpha_v)v(t-1) + u(t) \tag{30}$$

Such that:

$$\alpha_u = \frac{dt}{\tau_{\text{syn}}} \tag{31}$$
$$\alpha_v = \frac{dt}{\tau_{\text{mem}}} \tag{32}$$

With the constraints

$$w_{\text{in}}\frac{dt}{\tau_{\text{syn}}} = 1 \tag{33}$$
$$R\frac{dt}{\tau_{\text{mem}}} = 1 \tag{34}$$
$$v_{\text{leak}}\frac{dt}{\tau_{\text{mem}}} = 0 \tag{35}$$

Where the first constraint (w_{in}) can be hard-coded into a linear layer, or into the voltage threshold.

One may also implement v_{leak} through a bias term, but this was not done in our experiments.

Norse Norse discretizes the CuBa-LIF model as follows:

$$u(t+1) = u(t) - \frac{dt}{\tau_{syn}} i(t) \tag{36}$$

$$v(t+1) = v(t) + \frac{dt}{\tau_{mem}} \left(v_{leak} - v + u\right) \tag{37}$$

This deviates from the canonical formulation by lacking a weight and resistance term. The weight term is introduced by scaling the input, while the resistance term can be introduced by adding a weight factor between the synaptic (u) and somatic (v) parts.

snnTorch

$$u(t+1) = \alpha u(t) + i(t+1) \tag{38}$$

$$v(t+1) = \beta v(t) + u(t+1) \tag{39}$$

Leading to:

$$\alpha = 1 - \frac{dt}{\tau_{\text{syn}}} \tag{40}$$

$$\beta = 1 - \frac{dt}{\tau_{\text{mem}}} \tag{41}$$

With constraints:

$$w_{\text{in}} \frac{dt}{\tau_{\text{syn}}} = 1 \tag{42}$$

$$R \frac{dt}{\tau_{\text{mem}}} = 1 \tag{43}$$

$$v_{\text{leak}} \frac{dt}{\tau_{\text{mem}}} = 0 \tag{44}$$

Where a linear layer can be used with:

$$W_{ij} = \begin{cases} w_{\text{in}} \frac{dt}{\tau_{\text{syn}}} & \text{if } i = j \\ 0 & \text{otherwise} \end{cases} \tag{45}$$

to replace the first constraint.

snnTorch also offers a tunable membrane potential threshold which is shared across a population of neurons. As such, if W is a multiple of the identity matrix, the first constraint can also be satisfied by simply scaling the membrane potential threshold accordingly.

SpiNNaker2 In py-spinnaker2 [2] the CuBa-LIF model is given by:

$$I_{\text{syn}}(t) = \alpha_{syn} I_{\text{syn}}(t-1) + i(t) \tag{46}$$

$$v(t) = \alpha_{\text{mem}} v(t-1) + I_{\text{syn}}(t) + i_{\text{offset}} \tag{47}$$

As for LIF, two modes for the translation between NIR and SpiNNaker2 are supported:

In Exponential-Euler mode, the decay factors, the threshold and i_{offset} are calculated as follows:

$$\alpha_{\text{mem}} = \exp\left(-\frac{dt}{\tau_{\text{mem}}}\right) \tag{48}$$

$$\alpha_{\text{syn}} = \exp\left(-\frac{dt}{\tau_{\text{syn}}}\right) \tag{49}$$

$$\Theta = \theta_{\text{thr}} \left(\left(1 - \exp(-\frac{dt}{\tau_{\text{mem}}})\right) R \left(1 - \exp(-\frac{dt}{\tau_{syn}})\right) w_{\text{in}} \right)^{-1} \tag{50}$$

$$i_{\text{offset}} = \frac{v_{\text{leak}}}{R} \left(\left(1 - \exp(-\frac{dt}{\tau_{syn}})\right) w_{\text{in}} \right)^{-1} + i_{\text{bias}} \left(1 - \exp(-\frac{dt}{\tau_{syn}})\right)^{-1} \tag{51}$$

In Forward-Euler mode we match the discrete CuBa-LIF equations (36-37) so that SpiNNaker2 parameters become:

$$\alpha_{\text{mem}} = 1 - \frac{dt}{\tau_{mem}} \tag{52}$$

$$\alpha_{\text{syn}} = 1 - \frac{dt}{\tau_{syn}} \tag{53}$$

$$\Theta = \theta_{\text{thr}} \frac{\tau_{mem}}{dtR} \frac{\tau_{syn}}{dtw_{in}} \tag{54}$$

$$i_{\text{offset}} = \frac{v_{\text{leak}}}{R} \frac{\tau_{syn}}{dtw_{in}} + i_{\text{bias}} \frac{dt}{\tau_{syn}} \tag{55}$$

We note that in both modes the integration of the bias into i_{offset} is not exact. Instead, the conversion is chosen such that for bias-only input the membrane voltage converges to the same value as in the NIR model.

Rockpool The Rockpool CuBa-LIF neuron is a time-discretized simulation of exponential LIF dynamics.

$$\begin{gathered}
I_{syn}[n] = I_{syn}[n-1] + S_{in}[n] + W_{rec}\mathbf{S_{out}}[n-1] \\
I_{syn}[n] = \beta I_{syn}[n] \\
V_{mem}[n] = \alpha V_{mem}[n-1] \\
V_{mem}[n] = V_{mem}[n] + I_{syn}[n] + b_j + \sigma\zeta[n] \\
V_{mem}[n] > V_{thr} \rightarrow S_{out}[n] = 1 \\
V_{mem}[n] = V_{mem}[n] - V_{thr}
\end{gathered}$$

where $\alpha =$ and $\beta = \exp(-dt/\tau_{syn})$. Here W_{rec} is the recurrent weight matrix for the layer of neurons; $\mathbf{S_{out}}[n]$ is the vector of spikes produced by the layer at timestep n; and other symbols are as in the Rockpool LIF equations above. We have neglected neuron indices for simplicity; all neurons have independent state variables and parameters.

Xylo The Xylo CuBa-LIF neuron is a time-discretized low-bit-depth integer logic neuron, simulating an approximation of exponential LIF dynamics using integer bit-shift decay circuitry. In the following, the state variables I_{syn} and V_{mem} are 16-bit signed integers; W_{rec} are 8-bit signed integers; d_{syn} and d_{mem} are unsigned integer bit-shift decay parameters approximating $d \approx \log_2(\tau/dt)$; V_{thr} and b are 16-bit signed integers; S_{in} and S_{out} are unsigned integer number of events per dt.

$$I_{syn}[n] = I_{syn}[n-1] + S_{in}[n] + W_{rec}\mathbf{S_{out}}[n-1]$$

$$I_{syn}[n] = \begin{cases} I_{syn}[n] - I_{syn}[n] >> d_{syn} & \text{if } I_{syn}[n] >> d_{syn} > 0 \\ I_{syn}[n] - 1 & \text{otherwise} \end{cases} \tag{56}$$

$$V_{mem}[n] = \begin{cases} V_{mem}[n] - V_{mem}[n] >> d_{mem} & \text{if } V_{mem}[n] >> d_{mem} > 0 \\ V_{mem}[n] - 1 & \text{otherwise} \end{cases} \tag{57}$$

$$\begin{gathered}
V_{mem}[n] = V_{mem}[n] + I_{syn}[n] + b + \sigma\zeta[n] \\
V_{mem}[n] > \theta_{thr} \rightarrow S_{out}[n] = 1 \\
V_{mem}[n] = V_{mem}[n] - t\theta_{thr}
\end{gathered}$$

where $x >> n$ is the right-shift operator, shifting x by n bits.

The bit-shift decay operation in Eqs. 56 and 57 approximates exponential decay for V_{mem} and I_{syn} state variables. When the integer quantised decay parameters d_* obtained from τ_* are close to the corresponding floating-point values for d_*, this approximation causes only small deviations in dynamics. For values of V_{mem} and I_{syn} close to zero, where the bit-shift decay operation would not result in a decrease of the state variables, we include a unit decay per dt. We have neglected neuron indices in the above equations; all neurons have independent state variables and parameters.

B Relation to other intermediate representation and compilation frameworks

To clarify the relation of NIR to other compilers, code generators, optimizers, and network modelling tools, we provide the following comparison table. Most of the frameworks in the table solve different problems. As such, the table provides high-level and independent descriptions of each framework to situate NIR in the literature.

Supplementary Table 1: Comparison of ONNX, MLIR, LLVM, PyNN, NeuroML.

**Feature / Framework**	**ONNX (Open Neural Network Exchange)**	**MLIR (Multi-Level Intermediate Representation)**	**LLVM**	**PyNN**	**NeuroML**	**NIR**
**Scope**	AI model formats and runtimes	Compiler infrastructure targeting CPUs, GPUs, and more	Compiler for the optimization and generation of machine code	Abstraction layer for neural network descriptions, focusing on spiking models	Models neural connectivity and dynamics, focusing on biological realism	Declaration of discrete- or continuous-time neural primitives
**Language**	Not applicable (format)	Based on LLVM, supports many frontends (e.g., C++, Python)	Primarily C++	Python	XML-based description	Not applicable (format)
**Supported Platforms / Frameworks**	TensorFlow, PyTorch, and other major AI frameworks	Integrates with TensorFlow, PyTorch, and others through LLVM	Wide range of hardware from desktops to embedded devices	NEURON, Brian, NEST	NEURON, Brian, jLEMS	Lava, Loihi, Nengo, Norse, Rockpool, Sinabs, snnTorch, SpiNNaker2, Speck, Spyx, Xylo
**Typical Use Cases**	Exchanging models between different AI frameworks	Optimizing performance of machine learning models at compile time	Low-level optimization of applications in various programming languages	Research and educational use in computational neuroscience	Detailed simulations of neuronal behavior and neural circuitry for research	Application-dependent models and also able to exchange models between platforms

References

[1] Wulfram Gerstner, Werner M. Kistler, Richard Naud, and Liam Paninski. Neuronal Dynamics: From Single Neurons to Networks and Models of Cognition. Cambridge University Press, Cambridge, 2014.

[2] Bernhard Vogginger, Florian Kelber, Matthias Jobst, Yexin Yan, Pascal Gerhards, Martin Weih, and Mahmoud Akl. py-spinnaker2, November 2023. https://doi.org/10.5281/zenodo.10202110.
